# Long-term trajectories of mental health in Australia through COVID-19: Assessing distress and quality of life in priority populations

**DOI:** 10.1177/10398562251372821

**Published:** 2025-09-02

**Authors:** James Peak, Denny Meyer, Antonio Mendoza Diaz, Philip Sumner, Tamsyn Van Rheenen, Andrea Phillipou, Erica Neill, Wei Lin Toh, David Castle, Susan Rossell

**Affiliations:** Tasmanian Centre for Mental Health Service Innovation, 3917 Tasmanian Health Service, Hobart, TAS, Australia; School of Psychological Sciences, 3925 University of Tasmania, Hobart, TAS, Australia; Centre for Mental Health and Brain Sciences, 3783 Swinburne University of Technology, Melbourne, VIC, Australia; Tasmanian Centre for Mental Health Service Innovation, 3917 Tasmanian Health Service, Hobart, TAS, Australia; School of Psychological Sciences, 3925 University of Tasmania, Hobart, TAS, Australia; Centre for Mental Health and Brain Sciences, 3783 Swinburne University of Technology, Melbourne, VIC, Australia; Centre for Mental Health and Brain Sciences, 3783 Swinburne University of Technology, Melbourne, VIC, Australia; Department of Psychiatry, Melbourne Medical School, 276235 University of Melbourne, Melbourne, VIC, Australia; Centre for Mental Health and Brain Sciences, 3783 Swinburne University of Technology, Melbourne, VIC, Australia; Department of Mental Health, 60078 St Vincent’s Hospital, Melbourne, VIC, Australia; 276235 Orygen, Melbourne, VIC, Australia; Centre for Youth Mental Health, University of Melbourne, Melbourne, VIC, Australia; 276235 Orygen, Melbourne, VIC, Australia; Centre for Youth Mental Health, University of Melbourne, Melbourne, VIC, Australia; Centre for Mental Health and Brain Sciences, 3783 Swinburne University of Technology, Melbourne, VIC, Australia; Department of Mental Health, 60078 St Vincent’s Hospital, Melbourne, VIC, Australia; Tasmanian Centre for Mental Health Service Innovation, 3917 Tasmanian Health Service, Hobart, TAS, Australia; School of Medicine, 3925 University of Tasmania, Hobart, TAS, Australia; Centre for Mental Health and Brain Sciences, 3783 Swinburne University of Technology, Melbourne, VIC, Australia; Department of Mental Health, 60078 St Vincent’s Hospital, Melbourne, VIC, Australia

**Keywords:** COVID-19, quality of life, psychological distress, mental health, priority population

## Abstract

**Purpose:**

The COVID-19 pandemic significantly impacted mental health, particularly in individuals with pre-existing conditions and in younger people. However, long-term mental health trajectories beyond the acute phase remain unclear. This study examined distress levels and quality of life (QoL) in individuals with mental and/or physical health conditions, and across different age groups between 2020 and 2024.

**Methods:**

Using data from the COLLATE project – a series of online mental health surveys of the Australian public – we conducted general linear models to assess demographic parameters and risk factors associated with distress and QoL in 2,134 participants.

**Major Findings:**

Mental and physical health conditions were associated with higher distress and lower QoL throughout 2020 to 2024. Having both conditions had a compounding effect on QoL but not distress, which was primarily influenced by mental health conditions. Younger people reported higher distress across this period. Overall, QoL was lower in 2024 than 2020.

**Conclusion:**

Poorer mental health in those with mental and physical health conditions, and younger age groups, persisted from 2020 to 2024. This is concerning considering extensive literature demonstrating disproportionately greater impacts of COVID-19 on the mental health of priority populations and highlights groups that may require ongoing psychosocial support.

At the time of writing, Australia is approaching 5 years since the COVID-19 pandemic began. At the end of 2023, COVID-19 had caused 17,673 deaths in Australia, with the elderly and those with underlying medical conditions the most susceptible to severe illness and death.^1^ Beyond the acute somatic effects of COVID-19, the experience of the pandemic, and the government-mandated lifestyle changes (e.g. social restrictions) significantly impacted mental health^2–4^ and were widely documented in the early stages,^5^ but there remains limited data reflecting longer-term trends.

Individuals with mental or physical health conditions were particularly susceptible to poor mental health outcomes early in the pandemic. This included quality of life (QoL),^6–8^ psychological distress,^9,10^ and anxiety and depressive symptoms.^6,9,11^ Younger people also fared poorly; in Australia, levels of negative emotion were significantly greater in 2020 relative to Australian norms.^10^ Between 2019 and July 2020, the prevalence of psychological distress in Australia increased from 6% to 18%, while in younger people it increased from 8% to 30%.^12^ Similar trends were documented in the United Kingdom,^13^ United States,^14^ and Israel.^15^

Mental health impacts from pandemics and other disasters can persist long term and recovery patterns are influenced by demographics.^16^ For COVID-19, a return to pre-pandemic levels after an initial deterioration was reported,^14,17^ but substantial evidence from multiple countries (e.g. United States, Finland, and Singapore) points to sustained impacts on depression, anxiety, well-being, and resilience.^18–20^ There remains a lack of data tracking longer-term psychosocial impacts of COVID-19 on mental health in Australia, particularly in priority populations who were most impacted in the early stages.

We are now able to assess this in Australia using data from the COLLATE project (**CO**VID-19 and you: menta**L** hea**L**th in **A**us**T**ralia now surv**E**y), which consistently assessed the mental health of individuals living in Australia between 2020 and 2024. Here, we examined long-term trends in psychological distress and QoL in people reporting a mental or physical health condition, as well as investigating the influence of age. We predicted sustained poorer outcomes in these measures, that is, higher distress and lower QoL in people with mental or physical health conditions, and in younger people.

## Methods

### Study design

This study was based in Victoria, Australia, and approved by the Swinburne University Human Research Ethics Committee and complied with the Declaration of Helsinki. Participants were adults (18+) living in Australia who completed anonymous ∼15–20-min online surveys for the COLLATE project.^4^ Written informed consent was obtained. Data included were from May 2020 and April 2021–2024. These timepoints allowed for consistency in month of delivery across years. Recruitment occurred via digital and community noticeboards, social media, and participant registries held within Swinburne University, including participants with identified mental health conditions. Snowball sampling was used, with participants asked to share invitations. From the second year, recruitment also involved Prolific, filtered for Australian residents. A serial cross-sectional design was used, with different respondents per survey; only the last completed survey of participants with multiple responses was retained. Surveys covered a broad range of quantitative and qualitative mental health related questions, with only those relevant to this paper described below.

### Measures

#### Demographic and risk factors

The study included the demographic factors of age, gender, state of residence, and education level (Table 1). The primary risk factor was participants’ self-reported mental or physical health condition, assessed by indicating mental health diagnoses and confirming (yes/no) the presence of a current physical health condition.

**Table 1.** Sample characteristics.

Characteristic	May-20		Apr-21		Apr-22		Apr-23		Apr-24		Total	
	*N*	%	*N*	%	*N*	%	*N*	%	*N*	%	*N*	%
Total sample	528	100	265	100	350	100	558	100	456	100	2157	100
Gender												
Male	88	16.7	101	38.1	113	32.3	202	36.2	166	36.4	670	31.1
Female	426	80.7	160	60.4	228	65.1	348	62.4	279	61.2	1441	66.8
Self-described #	14	2.7	4	1.5	9	2.6	8	1.4	11	2.4	46	2.1
Crosstab test	X^2^ (4) = 69.52, Cramer’s V = 0.18, *p* < .001											
State												
Victoria	399	75.6	117	44.2	148	42.3	202	36.2	180	39.5	1046	48.5
Other	129	24.4	148	55.8	202	57.7	356	63.8	276	60.5	1111	51.5
Crosstab test	X^2^ (4) = 210.97, Cramer’s V = 0.31, *p* < .001											
Age group (years)												
18–24	31	5.9	51	19.2	70	20.0	111	19.9	61	13.4	324	15.0
25–29	74	14.0	58	21.9	60	17.1	111	19.9	92	20.2	395	18.3
30–34	76	14.4	51	19.2	50	14.3	100	17.9	77	16.9	354	16.4
35–39	75	14.2	38	14.3	35	10.0	77	13.8	54	11.8	279	12.9
40–49	111	21.0	30	11.3	60	17.1	74	13.3	91	20.0	366	17.0
50–59	89	16.9	20	7.5	44	12.6	48	8.6	44	9.6	245	11.4
60+	72	13.6	17	6.4	31	8.9	37	6.6	37	8.1	194	9.0
Crosstab test	X^2^ (24) = 123.89, Cramer’s V = 0.12, *p* < .001											
Education												
Primary/secondary school	53	10.0	46	17.4	57	16.3	93	16.7	59	12.9	308	14.3
TAFE/trade/diploma	102	19.3	35	13.2	48	13.7	85	15.2	74	16.2	344	15.9
Undergraduate	227	43.0	112	42.3	165	47.1	237	42.5	203	44.5	944	43.8
Postgraduate	146	27.7	72	27.2	80	22.9	143	25.6	120	26.3	561	26.0
Crosstab test	X^2^ (12) = 21.97, Cramer’s V = 0.12, *p* = .038											
Health condition												
No condition	264	50.0	146	55.1	188	53.7	294	52.7	262	57.5	1154	53.5
PH only	132	25.0	49	18.5	70	20.0	116	20.8	81	17.8	448	20.8
MH only	47	8.9	29	10.9	47	13.4	55	9.9	43	9.4	221	10.2
Both MH & PH	85	16.1	41	15.5	45	12.9	93	16.7	70	15.4	334	15.5
Crosstab test	X^2^ (12) = 17.13, Cramer’s V = 0.05, *p* = .145											

Note. MH = mental health; PH = physical health. #, self-described gender was not included in the crosstab test due to small sample size.

#### Psychological distress

Psychological distress was measured using the 21-item *Depression Anxiety Stress Scale* (DASS-21) (Cronbach’s standardized α: 0.85–0.96).^21^ It includes three sub-scales: depression, anxiety, and stress, each with seven items. Example questions are as follows: depression – ‘I felt that I had nothing to look forward to’; anxiety – ‘I felt I was close to panic’; stress – ‘I found myself getting agitated’. Items were scored on a 4-point (0 to 3) Likert scale. DASS-21 raw scores were doubled for comparability to the full length DASS scores (42-item) and underwent square root transformation to meet homoscedasticity and normality requirements of analyses. Higher scores indicate greater psychological distress.

#### Quality of life (QoL)

QoL was assessed using the *European Health Interview Surveys-Quality of Life* (EUROHIS-QoL) (Cronbach’s standardized α: 0.88–0.90),^22^ an eight-item measure on a 5-point Likert scale. Examples of questions include the following: ‘How would you rate your quality of life?’ and ‘How satisfied are you with your health?’. Higher scores indicate better perceived QoL.

#### Participant concerns

Participants ranked their top 10 COVID-19-related concerns (Table S1). The survey expanded from 23 options in 2020 to 36 options in 2024, retaining all original choices. Topics covered health and well-being (of self, family, and society), social distancing, the economy, medical care access, travel restrictions, and media coverage.

### Data and statistical analyses

Data were analysed in SPSS v29.0 with Bonferroni corrections for post-hoc tests. Complete case analysis was used, excluding participants with missing data. Raw data were collapsed into categories for reporting and analysis. Gender had three categories (male; female; self-described), but only male and female were included in general linear model analyses due to sample size imbalances. State was organized into two categories as substantially more participants were recruited from Victoria (Victoria; Other (NSW, QLD, SA, WA, ACT, NT, and TAS)). Age was grouped into seven categories (18–24; 25–29; 30–34; 35–39; 40–49; 50–59; 60+), and education into four categories (primary/secondary school; TAFE/trade/diploma; undergraduate; postgraduate). Health conditions were classified as No condition, PH (physical health) only, MH (mental health) only, and both MH & PH. Changes in sample characteristics over time were assessed using Crosstab tests (chi-squared test of association).

#### DASS-21 and EUROHIS-QOL

Univariate general linear modelling examined relationships between demographic variables (gender, state, age, education), year (2020–2024), and health condition (No condition, PH only, MH only, and both MH & PH), with psychological distress and QoL. Significant between-subjects effects were followed up with post-hoc tests. Bonferroni adjusted α-values for multiple comparisons are as follows: *p* < .008 for health condition, *p* < .004 for age group, and *p* < .013 for year.

#### Participant concerns

Each participant’s top ranked concern was assigned a value of 10, decreasing to 1 for the 10^th^ ranked concern; concerns outside of the top 10 for each participant were assigned a value of 0. We calculated the mean and standard deviation (SD) of all concerns across health condition and age groups and ranked concerns by means. We compared concerns from May 2020 and April 2024 and calculated a change score (ranking change). Negative scores indicated lower concern (relative to others) in 2024, and positive scores indicated higher concern in 2024.

## Results

### Sample description

Of 2,676 survey responses, 2,157 were eligible for inclusion in analyses, excluding those with incomplete data and multiple survey responses from a single participant (only their last survey was retained). The sample (Table 1) contained a higher proportion of female responses and those residing in Victoria, particularly in the first survey. A high proportion of respondents (69.8%) had a university-level education. Younger participants were more prevalent in later surveys. For health condition, 53.5% had no health condition, 20.8% had a physical but not a mental health condition, 10.2% had a mental but not a physical health condition while 15.5% had both. Table S2 shows the 10 most reported mental health conditions (accounting for ∼90% of all), with depression (24.4%), generalized anxiety disorder (21.6%), and social anxiety disorder (18.1%) the most common.

### Outcome measures

DASS-21 and EUROHIS-QoL scores by health condition and age group are presented in Tables S3 and S4, respectively. The proportion of participants in each DASS severity category in the first (2020) and last (2024) survey are provided for health condition and age groups in Tables S5 and S6, respectively. Table 2 shows significant (*p* < .05) predictors explaining more than 1% of the variation in the outcome measures (η^2^ > 0.01), before and after adding the health condition variable. Demographic factors and year together accounted for 8% of the variation in DASS-21 scores and 6.4% in EUROHIS-QoL scores. Adding in the health condition variable explained a further 18.2% of the variation in DASS-21 scores and 16.1% in EUROHIS-QoL scores.

**Table 2.** Significant predictors of psychological distress and QoL.

Outcome measure	Stage	Variables	*R*- square %	Significant predictors (η^2^)
Psychological distress	1	Demographics + year	8.0%	Age (0.054)
				Education (0.016)
	2	Health condition	26.2%	Age (0.019)
				Health condition (0.151)
QoL	1	Demographics + year	6.4%	Age (0.013)
				Education (0.042)
	2	Health condition	22.5%	Education (0.033)
				Health condition (0.139)

Note. Significant (*p* < .05) predictors of psychological distress and QoL with partial effect sizes (η^2^) > 0.010 are shown. *R*-square are adjusted values.

#### Psychological distress

Age (*F* (6, 2038) = 6.45, *p* < .001) and education (*F* (3, 2038) = 6.04, *p* < .001) were significantly associated with transformed DASS-21 scores (Table S7). Post-hoc tests showed that the 18–24-year-old group had significantly higher distress than age groups over 30 years, whilst the 60+ years group had lower distress than those aged under 50. There was no significant age by year interaction.

**Figure 1.**
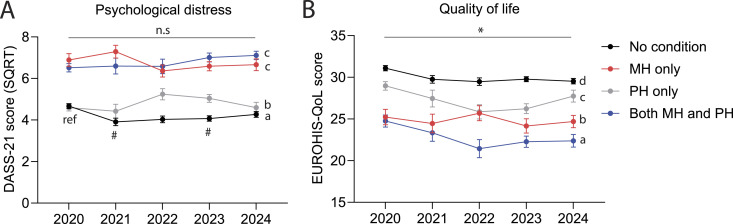
Mental health outcomes in each health condition group at different stages of the pandemic. (a) Mean (±SEM) transformed DASS-21 scores for each health condition group at each survey timepoint. (b) Same as A but for EUROHIS-QoL scores. _*_, *p* < .05; #, *p* < .013; a, b, c, and d, denote significant (*p* < .008) differences between groups; ‘ref’ indicates reference datapoint for post-hoc tests.

Figure 1(a) shows distress levels at each survey by health condition group (No condition, PH only, MH only, and both MH & PH). Health condition had a significant main effect on distress (*F* (3, 2038) = 120.48, *p* < .001). Post-hoc tests demonstrated that ‘No condition’ had significantly lower distress than all other groups. ‘PH only’ had significantly lower distress than ‘MH only’ and ‘both MH & PH’. There was no difference between ‘MH only’ and ‘both MH & PH’. There was no significant main effect of survey year, but a significant health condition × year interaction (*F* (12, 2038) = 3.51, *p* < .001) showed that distress was lower in the ‘No condition’ group in 2021 and 2023, relative to 2020 (2020 vs 2021, *p* = .004; 2020 vs 2023, *p* = .007).

#### QoL

**Figure 2.**
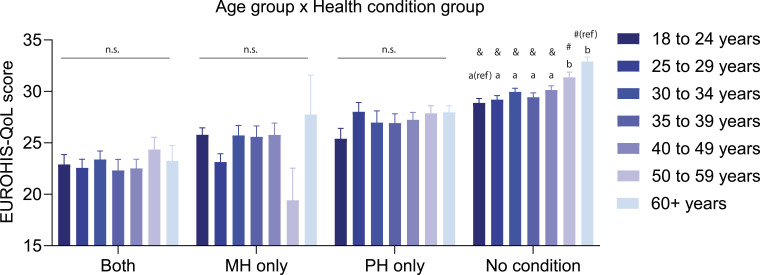
QoL for different age groups in each health condition group. Mean (±SEM) EUROHIS-QOL scores in each health condition group, split by age groups. ‘ref’ indicates reference group for two separate sets of post-hoc tests. ‘a’ and ‘b’ indicate non-significant or significant (*p* < .004) differences compared to the reference group (18–24 years), respectively. ‘#’ and ‘&’ indicate non-significant or significant (*p* < .004) differences compared to the reference group (60+ years).

State (*F* (1, 2038) = 6.25, *p* = .012) and education (*F* (3, 2038) = 23.36, *p* < .001) were significantly associated with QoL (Table S8). There was a main effect of year (*F* (4, 2038) = 2.87, *p* = .022), and post-hoc tests showed that QoL was significantly higher in 2020 than it was in later years. There was a main effect of health condition on QoL (Figure 1(b), *F* (3, 2038) = 109.51, *p* < .001), and post-hoc tests showed that QoL was significantly highest in group ‘No condition’ and lowest in group ‘both MH & PH’. Additionally, ‘MH only’ had significantly lower QoL than the ‘PH only’ group. There was a significant age group by health condition interaction (Figure 2; *F* (18, 2038) = 2.12, *p* = .004). Post-hoc tests compared the youngest (18–24 years) and oldest (60+ years) groups with each of the other age groups, within each health condition group. In the ‘no condition’ group, 18–24-year-olds had significantly lower QoL than those aged over 50, while the 60+ year olds had a significantly higher QoL than those aged 40–49 years and below. QoL did not differ by age in the other health condition groups.

#### Participant primary concerns

Table S9 lists the top 10 concerns for each health condition group in the 2020 and 2024 surveys. Ranking order was generally consistent across groups, with all groups ranking loved ones’ health and well-being highest. Changes in ranking order between 2020 and 2024 were mostly consistent across groups; concerns around social isolation and the implications of COVID on societal health and well-being were higher in 2020, while personal finances was higher in 2024. ‘Ongoing/persistent symptoms after having COVID-19’ ranked in the top 10 for all health condition groups except for the ‘No condition’ group.

Table S10 shows the top 10 concerns in 2020 and 2024 for the youngest (18–24 years) and oldest (60+ years) age groups. Both age groups consistently prioritized loved ones’ health. Younger people were more concerned about social isolation, personal finances, and employment, while older people were more concerned about COVID-19’s long-term health effects. ‘Catching COVID-19 myself’ was the highest ranked concern in 2024 for those aged over 60 years.

## Discussion

The COLLATE project, using a serial cross-sectional design, has tracked mental health and well-being in Australia since early in COVID-19, and it is a valuable resource to assess long-term mental health trajectories. Using this, we report on what is to our knowledge the longest period of COVID-19-related data captured in Australia to date. We found that from May 2020 to April 2024, psychological distress remained largely unchanged, and QoL declined. This is concerning, as early pandemic research showed poorer mental health relative to pre-pandemic levels. Our findings show that these effects, potentially induced by the pandemic, may have persisted, particularly in people with mental and physical health conditions and in younger people.

### Health conditions on psychological distress and QoL

Having a mental or a physical health condition was associated with higher distress, with the presence of a mental health condition the primary driver. This aligns with prior research showing higher distress among individuals with pre-existing conditions early in COVID-19, including those with suspected diagnoses^7^ and may be related to a greater susceptibility of those with mental health conditions to overestimate threat in conjunction with more difficulty accessing mental health support.^23^ In Australia in April 2020, distress was three times higher than Australian norms in those without a mental health history and over five times higher in those with a mental health history.^10^ Our findings show that this gap has endured until 2024.

Mental and physical health conditions were associated with poorer QoL, with comorbidity of both conditions compounding this effect. Our data confirm the persistence of poorer QoL in the early stages of the pandemic in people with mental health conditions and expand this to those with physical health conditions. COVID-19-related events (e.g. social isolation, illness, and life disruption) have been linked to lower QoL.^6^ As COVID-19 continues to cause illness and life disruption in 2024, this may help to explain the enduring effects. Our primary concerns analysis did not identify any stand-out concerns between health condition groups, and thus sustained impacts may reflect a multitude of factors, and this warrants further investigation. In fact, there were sustained concerns in all groups around COVID-19’s impacts on one’s self and loved-ones in 2024, while individuals with mental or physical health conditions held greater concern about ongoing symptoms of COVID-19.

### Age on psychological distress

We found that younger age was associated with higher distress that has persisted from 2020 to 2024, aligning with recently published data in the United States.^18^ The prevalence of distress in young Australians has increased over the past decade^24^ and the pandemic may have accelerated this. Age-related differences in anxiety and depression widened after pandemic onset,^12,14^ with a third of Australians aged 18–24 years showing consistently high levels of anxiety and depression in the first two years.^25^ Our data indicates scant improvement across all age groups over four years, which is particularly concerning for younger people.

It has been suggested that disruptions to crucial life-phases in younger people impacted social and romantic connection, job loss, and financial independence and may have hindered recovery in the pandemic context.^18^ Here, we found that younger people ranked concerns about social isolation, personal finances, and unemployment higher than older people. Social isolation worsened pandemic-related stress in 2020 and was reported more in younger people,^26^ while poor psychological well-being was linked with job loss and financial stress,^2,10,27^ possibly contributing to elevated distress among young people during COVID-19. Interestingly, older adults – despite being at a higher risk of severe COVID-19 – did not exhibit pronounced elevations in distress, even though they were most concerned about contracting COVID-19 in our 2024 data. Similar findings have been observed in American adults,^28^ and socio-emotional selectivity theory^29^ proposed as a possible explanation, as older adults may optimize emotional well-being when facing heightened mortality risk. Older adults’ life experience and past challenges may also enhance resilience against negative mental health effects in scenarios of high stress.^30^

### Limitations

Firstly, the COLLATE project’s recruitment methods resulted in a sample most representative of individuals who are highly educated, female, and residing in Victoria and may not be completely generalizable to the Australian population. Due to the online nature of the survey, individuals with limited internet access or low digital literacy were likely underrepresented. Secondly, while we included data matched for month of year to control for seasonal effects, the timeline of COVID-19 waves and government restrictions were more heterogenous, and a detailed monthly analysis could provide more temporal specificity regarding mental health trajectories. Thirdly, there was the lack of directly comparable pre-pandemic data, although early pandemic literature was extensive and supports our long-term findings. As our study was cross-sectional, clearer trends may emerge by tracking the same individuals over time. While not a limitation per se, the COVID-19 pandemic is not the only stressor in 2024, and the global context should be considered; inflationary and cost-of-living pressures have elevated in recent years, while geopolitical tensions and conflicts continue to escalate and may differentially impact people’s mental health.

### Conclusions

This study examined long-term mental health outcomes in priority populations over four years since early in COVID-19, demonstrating that adverse effects have persisted. Australian adults remain concerned about COVID-19’s health, social, and financial impacts. It highlights that people with mental and physical health conditions are susceptible to sustained negative mental health outcomes and that younger people have been consistently more distressed. The data are of interest to public health and pandemic preparedness and should be noted by policymakers in mental health, as it identifies priority populations in need of sustained resource allocation.

## Supplemental Material

Supplemental Material - Long-term trajectories of mental health in Australia through COVID-19: Assessing distress and quality of life in priority populations Supplemental Material for Long-term trajectories of mental health in Australia through COVID-19: Assessing distress and quality of life in priority populations by James Peak, Denny Meyer, Antonio Mendoza Diaz, Philip Sumner, Tamsyn Van Rheenen, Andrea Phillipou, Erica Neill, Wei Lin Toh, David Castle, and Susan Rossell in Australasian Psychiatry.

## Data Availability

The dataset is available on request by qualified researchers to the corresponding author. Requests require a proposal describing the purpose of data access, appropriate ethics approval, and data management plans.*

## Authors’ note

Susan Rossell is also affiliated with InsideOut Institute, University of Sydney, Sydney Local Health District, Sydney, NSW 2000, Australia.
