# Supplementary material for: Long-term trajectories of mental health in Australia through COVID-19: Assessing distress and quality of life in priority populations
Source: Australas Psychiatry. 2025 Sep 2;33(6):923–9. doi: 10.1177/10398562251372821 (PMC12657643; doi:10.1177/10398562251372821)
Supplement: Supplemental Material - Long-term trajectories of mental health in Australia through COVID-19: Assessing distress and quality of life in priority populations [file sj-pdf-1-apy-10.1177_10398562251372821.pdf]

Table S1: List of primary concerns presented to respondents in 2020 and 2024.

Question	2020	2024
1	Catching COVID-19 myself	Catching COVID-19 myself
2	Dying of COVID-19 myself	Dying of COVID-19 myself
3	Loved one catching COVID-19	Loved one catching COVID-19
4	Loved one dying from COVID-19	Loved one dying from COVID-19
5	Balancing work & caring for children/dependents	Balancing work & caring for children/dependents
6	Risk of unemployment or reduced employment	Risk of unemployment or reduced employment
7	Implications for health and wellbeing of self	Implications for health and wellbeing of self
8	Implications for health and wellbeing of family/loved ones	Implications for health and wellbeing of family/loved ones
9	Implications for health and wellbeing of society	Implications for health and wellbeing of society
10	Not being able to attend regular place of worship	Not being able to attend regular place of worship
11	Government communication of key messages	Government communication of key messages
12	Social isolation and social distancing	Social isolation and social distancing
13	Access to appropriate medical care	Access to appropriate medical care
14	Travel restrictions	Travel restrictions
15	The rapidly changing landscape	The rapidly changing landscape
16	Australian economy	Australian economy
17	World economy	World economy
18	Media coverage of the pandemic	Media coverage of the pandemic
19	Availability of food and medicines	Availability of food and medicines
20	Domestic violence	Domestic violence
21	Adapting to working from home (e.g. IT/connectivity issues)	Adapting to working from home (e.g. IT/connectivity issues)
22	Personal finances	Personal finances
23	Others (please specify)	Others (please specify)
24		Lifting government restrictions
25		I have no concerns
26		Uptake of vaccine booster shots
27		People breaking the rules (e.g. not wearing a mask, protesting, etc.)
28		Vaccine hesitancy
29		The interaction between the Federal and State governments
30		The increase in government power (e.g. the pandemic bill)
31		Returning to a normal way of life (e.g. attending the workplace, public events, etc.)
32		Declining effectiveness of vaccines over time
33		Being locked out of daily activities (e.g. shopping, the workplace) due to vaccine status
34		Vaccine availability for children under 12
35		Ongoing/persistent symptoms after having COVID-19
36		Risk of reinfection

Table S2. Counts and percentages of the top 10 most reported mental health conditions.

Mental health condition	N	%
Depression	**323**	24.4
Generalised anxiety disorder	**285**	21.6
Social anxiety disorder	**238**	18.1
Post traumatic stress disorder	**94**	7.1
Panic disorder	**82**	6.3
Obsessive compulsive disorder	**67**	5.1
Body dysmorphic disorder	**39**	2.9
Attention deficit hyperactivity disorder	**28**	2.1
Binge eating disorder	**27**	2.0
Anorexia nervosa	**15**	1.1

Table S3: Outcome measures for each health condition group

	No condition (n=1154)		PH only (n=448)		MH only (n=221)		Both (n=334)	
	Mean	SD	Mean	SD	Mean	SD	Mean	SD
DASS-21								
Depression	7.9	8.3	10.0	9.7	17.8	11.4	18.6	11.8
Anxiety	4.6	5.9	5.7	6.6	12.4	8.5	12.8	9.0
Stress	10.1	7.9	11.6	7.9	18.1	9.0	18.9	9.1
Total	22.6	19.5	27.3	21.2	48.3	24.7	50.3	25.6
Quality of life	30.0	5.4	27.3	6.5	24.9	6.0	22.8	6.5

Table notes: MH = mental health; PH = physical health. Data include male, female, and self-described genders.

Table S4: Outcome measures for each age group (years)

	18-24 (n=324)		25-29 (n=395)		30-34 (n=354)		35-39 (n=279)		40-49 (n=366)		50-59 (n=245)		60+ (n=194)	
	Mean	SD	Mean	SD	Mean	SD	Mean	SD	Mean	SD	Mean	SD	Mean	SD
DASS-21														
Depression	13.6	11.0	12.8	11.2	10.5	9.9	11.2	10.4	10.1	10.1	8.7	9.6	8.0	9.2
Anxiety	9.9	8.6	8.8	8.6	6.5	7.0	6.8	7.7	6.0	7.0	4.6	5.9	3.5	5.2
Stress	14.8	9.4	14.2	9.4	12.1	8.2	13.0	9.0	12.9	9.0	10.1	8.0	8.6	7.3
Total	38.3	26.3	35.7	26.4	29.0	21.8	31.0	24.1	29.1	23.2	23.4	21.0	20.1	19.2
Quality of life	26.9	6.1	27.0	6.7	28.0	6.3	27.6	6.2	27.8	6.5	28.8	6.8	29.6	6.0

Table notes: Data include male, female and self-described genders.

Table S5: DASS severity ratings for each health condition group

	No condition		PH only		MH only		Both PH & MH	
	2020 (n=264)	2024 (n=262)	2020 (n=132)	2024 (n=81)	2020 (n=47)	2024 (n=43)	2020 (n=85)	2024 (n=70)
Depression								
Normal	61.0%	61.1%	60.6%	61.7%	23.4%	20.9%	24.7%	20.0%
Mild	15.9%	14.9%	15.9%	13.6%	8.5%	25.6%	15.3%	8.6%
Moderate	13.6%	14.5%	15.2%	12.3%	23.4%	23.3%	24.7%	34.3%
Severe	5.3%	4.6%	5.3%	1.2%	12.8%	11.6%	18.8%	12.9%
Extremely Severe	4.2%	5.0%	3.0%	11.1%	31.9%	18.6%	16.5%	24.3%
Anxiety								
Normal	77.7%	72.5%	75.8%	66.7%	34.0%	32.6%	38.8%	30.0%
Mild	6.4%	6.1%	4.5%	9.9%	8.5%	7.0%	14.1%	8.6%
Moderate	9.5%	13.4%	12.9%	8.6%	19.1%	27.9%	21.2%	20.0%
Severe	4.5%	3.8%	5.3%	6.2%	12.8%	11.6%	4.7%	12.9%
Extremely Severe	1.9%	4.2%	1.5%	8.6%	25.5%	20.9%	21.2%	28.6%
Stress								
Normal	70.8%	75.2%	71.2%	74.1%	36.2%	37.2%	41.2%	30.0%
Mild	11.0%	8.8%	18.2%	11.1%	17.0%	20.9%	16.5%	20.0%
Moderate	12.1%	10.3%	6.8%	11.1%	17.0%	20.9%	15.3%	24.3%
Severe	4.9%	4.6%	3.8%	1.2%	21.3%	11.6%	23.5%	14.3%
Extremely Severe	1.1%	1.1%	0.0%	2.5%	8.5%	9.3%	3.5%	11.4%

Table notes: MH = mental health; PH = physical health. Data include male, female, and self-described genders. Severity category cut-off values are those defined in Lovibond et al. (1993) (21).

Table S6: DASS severity ratings for each age group (years)

	18-24 years		25 to 29 years		30 to 34 years		35 to 39 years		40 to 49 years		50 to 59 years		60 years+	
	2020 (n=31)	2024 (n=61)	2020 (n=74)	2024 (n=92)	2020 (n=76)	2024 (n=77)	2020 (n=75)	2024 (n=54)	2020 (n=111)	2024 (n=91)	2020 (n=89)	2024 (n=44)	2020 (n=72)	2024 (n=37)
Depression														
Normal	41.9%	36.1%	45.9%	46.7%	47.4%	42.9%	46.7%	51.9%	52.3%	56.0%	59.6%	61.4%	61.1%	78.4%
Mild	9.7%	21.3%	10.8%	12.0%	17.1%	18.2%	13.3%	14.8%	18.0%	13.2%	13.5%	18.2%	19.4%	2.7%
Moderate	19.4%	24.6%	20.3%	22.8%	21.1%	20.8%	17.3%	16.7%	16.2%	16.5%	15.7%	9.1%	8.3%	5.4%
Severe	16.1%	4.9%	10.8%	8.7%	7.9%	9.1%	14.7%	5.6%	5.4%	6.6%	4.5%	0.0%	4.2%	0.0%
Extremely Severe	12.9%	13.1%	12.2%	9.8%	6.6%	9.1%	8.0%	11.1%	8.1%	7.7%	6.7%	11.4%	6.9%	13.5%
Anxiety														
Normal	48.4%	41.0%	56.8%	55.4%	76.3%	54.5%	56.0%	63.0%	68.5%	68.1%	70.8%	81.8%	80.6%	78.4%
Mild	12.9%	4.9%	5.4%	7.6%	3.9%	6.5%	9.3%	7.4%	8.1%	8.8%	7.9%	6.8%	6.9%	8.1%
Moderate	19.4%	16.4%	17.6%	17.4%	9.2%	26.0%	16.0%	9.3%	12.6%	13.2%	13.5%	4.5%	6.9%	8.1%
Severe	9.7%	9.8%	8.1%	8.7%	3.9%	6.5%	6.7%	7.4%	3.6%	4.4%	7.9%	0.0%	1.4%	5.4%
Extremely Severe	9.7%	27.9%	12.2%	10.9%	6.6%	6.5%	12.0%	13.0%	7.2%	5.5%	0.0%	6.8%	4.2%	0.0%
Stress														
Normal	58.1%	44.3%	55.4%	65.2%	56.6%	53.2%	54.7%	64.8%	57.7%	71.4%	73.0%	79.5%	84.7%	83.8%
Mild	6.5%	19.7%	9.5%	9.8%	19.7%	20.8%	18.7%	9.3%	17.1%	7.7%	16.9%	9.1%	4.2%	5.4%
Moderate	12.9%	23.0%	16.2%	14.1%	13.2%	18.2%	16.0%	9.3%	12.6%	11.0%	6.7%	4.5%	5.6%	10.8%
Severe	9.7%	6.6%	16.2%	6.5%	10.5%	3.9%	8.0%	11.1%	10.8%	7.7%	3.4%	4.5%	5.6%	0.0%
Extremely Severe	12.9%	6.6%	2.7%	4.3%	0.0%	3.9%	2.7%	5.6%	1.8%	2.2%	0.0%	2.3%	0.0%	0.0%

Table notes: Data include male, female and self-described genders. Severity category cut-off values used are those defined in Lovibond et al. (1993) (21).

Table S7: General Linear Model results for Psychological Distress

Variable	N	Marginal Means [95% CIs]	Std Error	*F*-value	*p*-value	η2
Gender				2.74	0.098	0.001
Male	670	5.44 [5.24 – 5.64]	0.10			
Female	1441	5.60 [5.44 – 5.76]	0.08			
State				0.00	0.955	0.000
Victoria	1017	5.52 [5.34 – 5.70]	0.09			
Other	1094	5.52 [5.35 – 5.70]	0.09			
**Age group (years)**				**6.45**	**<0.001**	**0.019**
18-24	314	6.04 [5.74 – 6.33]	0.15			
25-29	386	6.12 [5.86 – 6.38]	0.13			
30-34	343	5.70 [5.41 – 5.99]	0.15			
35-39	274	5.68 [5.35 – 6.00]	0.17			
40-49	359	5.44 [5.15 – 5.72]	0.15			
50-59	244	5.03 [4.57 – 5.49]	0.24			
60+	191	4.64 [4.02 – 5.25]	0.31			
**Education**				**6.04**	**<0.001**	**0.009**
Primary/secondary school	297	5.78 [5.51 – 6.05]	0.14			
TAFE/Trade/Diploma	340	5.67 [5.42 – 5.91]	0.12			
Undergraduate degree	918	5.43 [5.25 – 5.62]	0.09			
Postgraduate degree	556	5.20 [4.98 – 5.41]	0.11			
Year				0.92	0.453	0.002
2020	514	5.70 [5.45 – 5.95]	0.13			
2021	261	5.45 [5.11 – 5.79]	0.17			
2022	341	5.38 [5.09 – 5.67]	0.15			
2023	550	5.55 [5.32 – 5.79]	0.12			
2024	445	5.52 [5.26 – 5.79]	0.14			
**Health condition**				**120.48**	**<0.001**	**0.151**
Both	313	6.63 [6.35 – 6.90]	0.14			
MH only	213	6.46 [6.02 – 6.90]	0.22			
PH only	438	4.93 [4.71 – 5.15]	0.11			
No condition	1147	4.06 [3.92 – 4.20]	0.07			
**Year x Health condition**				**3.51**	**<0.001**	**0.020**
Age group x Health condition				1.45	0.100	0.013
Year x Age group				1.44	0.079	0.017

Table notes: Bold text indicate variables significantly associated with psychological distress.

Table S8: General Linear Model results for Quality of life

Variable	N	Marginal Means [95% CIs]	Std Error	F-value	p-value	η2
Gender				2.13	0.145	0.001
Male	670	25.79 [25.23 – 26.36]	0.29			
Female	1441	26.20 [25.76 – 26.65]	0.23			
**State**				**6.25**	**0.012**	**0.003**
Victoria	1017	26.33 [25.82 – 26.84]	0.26			
Other	1094	25.67 [25.17 – 26.16]	0.25			
Age group (years)				1.86	0.085	0.005
18-24	314	26.37 [25.53 – 27.21]	0.43			
25-29	386	25.04 [24.31 – 25.77]	0.37			
30-34	343	26.05 [25.24 – 26.87]	0.42			
35-39	274	25.72 [24.80 – 26.64]	0.47			
40-49	359	25.91 [25.10 – 26.71]	0.41			
50-59	244	25.35 [24.04 – 26.65]	0.67			
60+	191	27.55 [25.82 – 29.29]	0.89			
**Education**				**23.36**	**<0.001**	**0.033**
Primary/secondary school	297	24.86 [24.10 – 25.63]	0.39			
TAFE/Trade/Diploma	340	24.95 [24.27 – 25.64]	0.35			
Undergraduate degree	918	26.35 [25.84 – 26.87]	0.26			
Postgraduate degree	556	27.82 [27.21 – 28.43]	0.31			
**Year**				**2.87**	**0.022**	**0.006**
2020	514	26.95 [26.26 – 27.65]	0.36			
2021	261	26.03 [25.07 – 27.00]	0.49			
2022	341	25.58 [24.76 – 26.40]	0.42			
2023	550	25.56 [24.89 – 26.22]	0.34			
2024	445	25.87 [25.12 – 26.61]	0.38			
**Health condition**				**109.51**	**<0.001**	**0.139**
Both	313	22.79 [22.02 – 23.55]	0.39			
MH only	213	24.52 [23.28 – 25.75]	0.63			
PH only	438	26.84 [26.22 – 27.46]	0.32			
No condition	1147	29.85 [29.45 – 30.24]	0.20			
Year x Health condition				1.50	0.116	0.009
**Age group x Health condition**				**2.12**	**0.004**	**0.018**
Year x Age group				1.23	0.203	0.014

Table notes: Bold text indicate variables significantly associated with quality of life.

Table S9: Rank data illustrating the top 10 concerns of participants in each health condition group in 2020 and 2024.

	No condition					PH only					MH only					Both PH & MH				
	2020 (n=264)		2024 (n=262)		Change	2020 (n=132)		2024 (n=81)		Change	2020 (n=47)		2024 (n=43)		Change	2020 (n=85)		2024 (n=70)		Change
**Primary Concern**	Mean (SD)	Rank	Mean (SD)	Rank		Mean (SD)	Rank	Mean (SD)	Rank		Mean (SD)	Rank	Mean (SD)	Rank		Mean (SD)	Rank	Mean (SD)	Rank	
Implications for health and wellbeing of family/loved ones	5.91 (3.15)	1	3.13 (3.62)	2	-1	5.99 (3.04)	1	3.57 (3.67)	3	-2	5.09 (3.71)	2	3.23 (3.71)	2	0	5.18 (3.26)	2	3.60 (3.44)	3	-1
Loved one dying from COVID-19	5.36 (4.23)	2	3.62 (4.36)	1	+1	5.59 (4.21)	2	3.81 (4.20)	1	+1	5.47 (4.08)	1	5.16 (4.48)	1	0	5.53 (4.11)	1	4.23 (4.58)	1	0
Loved one catching COVID-19	4.87 (3.95)	3	3.10 (4.02)	3	0	5.09 (3.92)	3	2.91 (3.80)	5	-2	4.45 (3.75)	3	3.14 (3.91)	4	-1	4.67 (3.89)	4	3.96 (4.11)	2	+2
Implications for health and wellbeing of society	4.14 (3.31)	4		12	-8	4.06 (3.13)	5		12	-7	3.79 (3.20)	6		17	-11	3.78 (3.22)	6		11	-5
Implications for health and wellbeing of self	3.78 (3.34)	5	2.80 (3.52)	4	+1	4.41 (3.37)	4	3.58 (3.71)	2	+2	4.28 (3.38)	4	3.23 (3.62)	3	+1	5.12 (3.28)	3	3.53 (3.41)	4	-1
Social isolation and social distancing	3.68 (3.46)	6		15	-9	3.41 (3.24)	8		19	-11	3.96 (3.51)	5		15	-10	3.87 (3.56)	5		23	-18
Australian economy	3.57 (3.06)	7	2.33 (3.33)	5	+2	2.89 (2.80)	9	1.83 (3.15)	9	0	3.26 (2.99)	9	2.86 (3.37)	6	+3		11	2.03 (3.25)	10	+1
Risk of unemployment or reduced employment	3.16 (3.65)	8	1.73 (3.22)	10	-2	2.10 (3.29)	10		13	-3	3.70 (3.77)	7	2.28 (3.69)	9	-2	2.78 (3.36)	9	2.10 (3.24)	8	+1
Travel restrictions	2.78 (3.14)	9		14	-5															
Catching COVID-19 myself	2.56 (3.32)	10	2.17 (3.52)	8	+2	3.82 (3.87)	6	3.02 (3.97)	4	+2	3.43 (3.88)	8	2.77 (3.87)	7	+1	2.88 (3.89)	7	3.46 (3.98)	5	+2
Personal finances		12	2.27 (3.33)	6	+6		14	2.19 (3.45)	7	+7	3.13 (3.57)	10	2.95 (3.54)	5	+5	2.48 (3.51)	10	2.61 (3.64)	6	+4
Dying of COVID-19 myself		13	1.82 (3.40)	9	+4	3.54 (4.07)	7	2.74 (3.92)	6	+1		11	2.56 (4.01)	8	+3	2.69 (3.81)	8	2.04 (3.42)	9	-1
Access to appropriate medical care							NA	1.79 (2.81)	10	NA										
I have no concerns		NA	2.23 (4.15)	7	NA															
Ongoing/persistent symptoms after having COVID-19							NA	2.17 (3.36)	8	NA		NA	1.51 (2.91)	10	NA		NA	2.53 (3.60)	7	NA

Table notes: Primary concerns that received an NA in the rank or change column were additional questions from the original list of primary concerns in 2020. Values of zero were assigned to options not endorsed by a participant, and values of 10 to 1 were computed for concerns ranked from greatest concern. Red text in the change column indicates less concern in 2024 as compared to 2020 while green text indicates more concern. Grey values indicate instances where the concern fell outside of the overall top ranks (of all participants within a group) in either year (2020 or 2024) but was in the top 10 concerns in the alternative year (2020 to 2024).

Table S10: Rank data illustrating the top 10 concerns of participants in the youngest and oldest age groups.

	18-24 years					60+ years				
	2020 (*n*=31)		2024 (*n*=61)		Change	2020 (*n*=72)		2024 (*n*=37)		Change
**Primary Concern**	Mean (SD)	Rank	Mean (SD)	Rank		Mean (SD)	Rank	Mean (SD)	Rank	
Loved one dying from COVID-19	6.68 (4.03)	1	5.15 (4.63)	1	0	5.71 (4.11)	2	2.57 (3.66)	5	-3
Implications for health and wellbeing of family/loved ones	5.77 (3.08)	2	3.52 (3.61)	2	0	6.17 (2.89)	1	3.30 (3.67)	4	-3
Social isolation and social distancing	5.58 (3.14)	3		15	-12	3.33 (3.46)	8		20	-12
Implications for health and wellbeing of self	4.65 (3.37)	4	3.20 (3.47)	4	0	3.61 (3.31)	6	4.16 (3.90)	3	+3
Loved one catching COVID-19	4.35 (4.20)	5	3.23 (3.95)	3	+2	5.69 (4.09)	3	4.24 (4.32)	2	+1
Risk of unemployment or reduced employment	3.74 (3.55)	6	1.84 (3.39)	10	-4					
Implications for health and wellbeing of society	2.90 (3.10)	7		11	-4	4.22 (2.98)	5	2.30 (3.52)	6	-1
Travel restrictions	2.71 (3.06)	8		18	-10	2.24 (2.76)	10		27	-17
Australian economy	2.61 (3.01)	9	2.15 (3.25)	8	+1	3.49 (2.89)	7		15	-8
Catching COVID-19 myself	2.16 (3.23)	10	2.49 (3.59)	6	+4	4.25 (4.05)	4	4.92 (4.49)	1	+3
Personal finances		11	2.44 (3.41)	7	+4					
Dying of COVID-19 myself		12	2.51 (3.60)	5	+7	2.93 (4.02)	9	2.19 (3.68)	7	+2
I have no concerns		NA	1.89 (3.89)	9	NA					
Ongoing/persistent symptoms after having COVID-19							NA	2.03 (3.39)	8	NA
Uptake of vaccine booster shots							NA	1.95 (3.22)	9	NA
Vaccine hesitancy							NA	1.70 (2.64)	10	NA

Table notes: Primary concerns that received an NA in the rank or change column were additional questions from the original list of primary concerns in 2020. Values of zero were assigned to options not endorsed by a participant, and values of 10 to 1 were computed for concerns ranked from greatest concern. Red text in the change column indicates less concern in 2024 as compared to 2020 while green text indicates more concern. Grey values indicate instances where the concern fell outside of the overall top ranks (of all participants within a group) in either year (2020 or 2024) but was in the top 10 concerns in the alternative year (2020 to 2024).
